# The Association between Dietary Fiber Intake and Serum Klotho Levels in Americans: A Cross-Sectional Study from the National Health and Nutrition Examination Survey

**DOI:** 10.3390/nu15143147

**Published:** 2023-07-14

**Authors:** Si Liu, Mingyang Wu, Yan Wang, Lu Xiang, Gang Luo, Qian Lin, Lin Xiao

**Affiliations:** Xiangya School of Public Health, Central South University, Changsha 410078, China; 226911029@csu.edu.cn (S.L.); mingyangwu2016@163.com (M.W.); 226911039@csu.edu.cn (Y.W.); xianglu66@csu.edu.cn (L.X.); luogang@csu.edu.cn (G.L.); linqian@csu.edu.cn (Q.L.)

**Keywords:** dietary fiber, serum Klotho, National Health and Nutrition Examination Survey (NHANES), nutritional epidemiology

## Abstract

Background: Klotho is an aging-related marker closely associated with a number of diseases. A growing body of evidence suggests that dietary factors and lifestyle habits can impact serum Klotho levels. The effect of dietary fiber, a key component of a healthy diet, on the body’s serum Klotho levels has not been fully elucidated. Objective: The aim of this study was to explore the relationship between dietary fiber intake and serum Klotho levels in people aged 40–79 years in the United States. Methods: A total of 11,282 participants were included in this study, all from the National Health and Nutrition Examination Survey from 2007 to 2016. Dietary fiber intake was assessed by uniformly trained interviewers using the 24 h dietary recall method. Serum Klotho was quantified using commercially available ELISA kits manufactured by IBL International, Japan. The relationship between dietary fiber intake and serum Klotho levels was analyzed using a multiple linear regression model. Subsequently, the non-linear dose–response relationship between the two was further explored using a restricted cubic spline (RCS) model. Results: After adjusting for potential confounders, serum Klotho levels increased by 1.9% (95% confidence interval [CI]: 0.8%, 3.0%) for each interquartile range increase in dietary fiber intake in all participants. Considering dietary fiber intake as a categorical variable, serum Klotho levels were found to be 4.7% higher in participants in the highest quartile of dietary fiber intake than in those in the lowest quartile (95% CI: 1.8%, 7.6%). RCS plots depicted a non-linear positive correlation between dietary fiber intake and serum Klotho levels. Subgroup analysis revealed that the relationship between dietary fiber intake and serum Klotho levels was more pronounced in older (percentage change: 7.0%; 95% CI: 2.5%, 11.7%) and overweight and obese participants (percentage change: 4.9%; 95% CI: 1.5%, 8.4%). Conclusions: The results of this study showed that dietary fiber intake was significantly associated with serum Klotho levels in participants. This finding is yet to be further confirmed by prospective studies.

## 1. Introduction

Dietary fiber, a key component of a healthy dietary pattern [1], is by nature an indigestible carbohydrate [2]. Plant foods such as vegetables, fruits, nuts, grains, and legumes are rich sources of dietary fiber in our daily life [3]. Dietary fiber can reach the colon and thus influence intestinal physiology, and it plays a role in regulating intestinal microbiota [4]. It also possesses physiological functions including reduction of inflammatory response [5] and antioxidant [6]. Accumulating evidence indicates that dietary fiber intake is beneficial in alleviating several chronic non-communicable diseases, such as hypertension [7], diabetes [8], hyperlipidemia [9], and cancer [10]. Furthermore, dietary fiber has been demonstrated to have significant antiaging benefits, with people who consume high levels of dietary fiber living longer than their peers and having a reduced risk of disease or premature death [11]. In the United States, the recommended daily intake of dietary fiber for adults is 30–35 g for men and 25–32 g for women. However, more than 90% of women and 97% of men do not meet this recommended standard of dietary fiber intake [5,12].

The *Klotho* gene, for the first time, gained attention in an extremely prematurely mutated mouse. The absence of *Klotho* gene expression in mice led to a short lifespan and the degeneration of multiple organs [13]. The *Klotho* gene encodes the Klotho protein, a pleiotropic protein that includes three isoforms: α-, β-, and γ-Klotho. Klotho performs multiple physiological and pathological functions in several organs and tissues of the body [14]. The human liver and pancreas mainly express the β-Klotho protein, which is involved in bile acid and energy metabolism [15]. γ-Klotho is primarily expressed in the skin and kidney, and its specific function has not been elucidated heretofore [16]. The α-Klotho protein is a one-way transmembrane protein [17], mainly expressed in human kidney cells [18] and choroid plexus [19]. It can be cleaved by the ADAM family of metalloproteinases to release soluble α-Klotho (S-Klotho), which then enters the blood, urine, and cerebrospinal fluid to act [17]. This study focused on S-Klotho derived from α-Klotho in serum, hereafter abbreviated as Klotho.

Klotho does not only have recognized functions in antiaging, the regulation of phosphate homeostasis, and vitamin D metabolism [20,21]. Mounting evidence suggests that it is also closely associated with a variety of diseases such as Alzheimer’s disease [22], diabetes [23], atherosclerosis [24], and chronic kidney disease [25]. Klotho protein expression is now known to be influenced by a combination of factors. Its expression significantly decreases with age, both in animals and humans [26]. Fibroblast growth factor 23 or estrogen deficiency upregulates Klotho expression, whereas oxidative stress and angiotensin II inhibit Klotho expression [27]. Furthermore, systemic and local inflammation can decrease Klotho levels [28]. At the same time, dietary factors and lifestyle habits are highly associated with Klotho levels; for example, pro-inflammatory dietary patterns and smoking and alcohol consumption can lead to decreased Klotho levels [29,30,31]. High Mediterranean diet adherence favors elevated Klotho levels [32]. It is worth mentioning that dietary fiber has powerful anti-inflammatory and antioxidant protective effects [5,6], and it is also inextricably linked to a number of health outcomes such as aging [11,33]. In the nutritional dietary guidelines, dietary fiber is recommended as an integral component of a healthy diet [34]. However, Klotho level happens to be influenced by the inflammatory and oxidative stress status of the body [28,35]. It has been reported that there is an association between dietary fiber intake and serum Klotho levels [36]. However, in-depth epidemiological studies of the relationship between these two based on large population samples are sorely lacking, and they deserve further exploration.

Based on the extant literature, we hypothesized a positive correlation between dietary fiber intake and serum Klotho protein level. The relationship between the two was analyzed in a population using nationally representative data obtained from the US National Health and Nutrition Examination Survey (NHANES). The prime objective of this study was to expand on the nutritional epidemiological findings between dietary fiber intake and serum Klotho levels and to provide a scientific basis for future dietary guidance and health care.

## 2. Materials and Methods

### 2.1. Study Population Samples

This study is based on NHANES—the National Health and Nutrition Examination Survey—a nationwide cross-sectional survey conducted in the United States, including questionnaires conducted at home and standardized health examinations at specialized mobile examination centers. Its function is to monitor the health and nutritional status of adults and children in the United States. The NHANES is administered by the US National Center for Health Statistics (NCHS), which is affiliated with the Centers for Disease Control and Prevention. The survey began in 1960 and has been developed for more than half a century. Still being updated is the continuous NHANES, which takes place every two years. The NHANES is well represented and uses a multi-stage stratified probability sampling method [37], with about 5000 individuals participating in the survey each year. All participants in this survey signed written informed consent. At the same time, the NCHS Ethics Review Committee approved the investigation plan and research protocol. The official website of NHANES provides more comprehensive and detailed information (http://www.cdc.gov/nchs/nhanes.htm, accessed on 18 March 2023).

**Figure 1:**
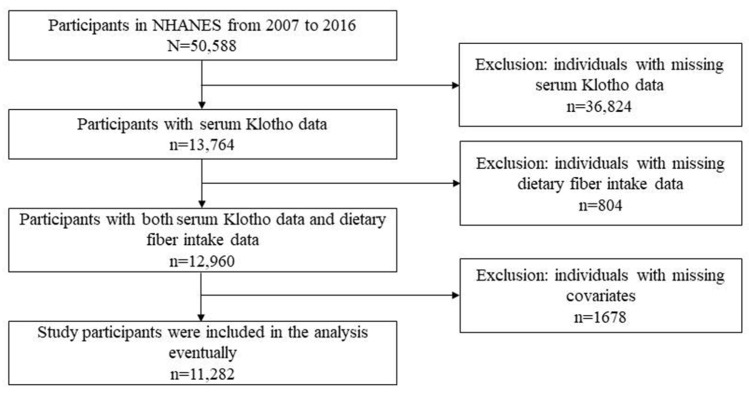
Flowchart of screening study participants. NHANES: the National Health and Nutrition Examination Survey.

Our study participants were all from the 2007–2016 cycle of the continuous NHANES, totaling 50,588 participants. Of those, 11,282 participants were eligible, meeting the inclusion criteria. We excluded participants with missing serum Klotho data (*n* = 36,824. (Serum Klotho levels were only measured in participants aged 40–79 years in the NHANES study. The age of the youngest participant in this study was 40 years old.) We also excluded those with lacking dietary fiber data at the first 24 h dietary review (*n* = 804) and those with incomplete covariates (age, sex, body mass index (BMI), poverty income ratio (PIR), educational attainment, ethnicity, serum cotinine, alcohol drinking, diabetes, hypertension, estimated glomerular filtration rate (eGFR), and dietary energy intake) (*n* = 1678). The detailed process of participant exclusion and inclusion is depicted in Figure 1.

### 2.2. Assessment of Dietary Fiber Intake

Dietary fiber intake was considered an independent variable in this study, and the unit of measurement was g/day. All NHANES participants were eligible to participate in the 24 h meal review interview. The first interview took place in the private room of NHANES MEC. A set of measurement guidelines, including various cups, bowls, spoons, measuring cups, rulers, etc., were provided in the MEC Dietary Interview Room for participants to report the amount of food consumed. The interviewers were trained rigorously and uniformly, and computer-aided interviews were conducted. Detailed dietary intake information was collected from study participants over a 24 h period (midnight to midnight). Each participant’s dietary fiber intake was calculated based on the US Department of Agriculture (USDA) Food and Nutrient Databases for Dietary Studies (FNDDS) (https://www.cdc.gov/nchs/tutorials/dietary/SurveyOrientation/ResourceDietaryAnalysis/intro.htm, accessed on 18 March 2023). Dietary fiber from different types of foods was determined according to food codes [38].

### 2.3. Determination of Serum Klotho Levels

The original blood samples used to measure serum Klotho levels were all from NHANES participants aged 40–79 who agreed to have their blood samples for follow-up studies. For the quantitative analysis of Klotho, we adopted commercially available ELISA kits manufactured by IBL International in Japan. The IBL ELISA method used to measure Klotho levels in serum samples was validated from multiple angles, and the validation results were made available to experimental investigators prior to the start of the formal study. All sample analyses were performed in duplicate according to the manufacturer’s protocol, and the average of the two tests was used to calculate the final value. At the same time, all results were checked to meet the laboratory standardized acceptability criteria before issuing the report. All pre-analyzed samples were stored at −80 degrees Celsius, and strict quality control was performed throughout the experiment [39].

### 2.4. Covariate Adjustment

The covariates adjusted for this study included age, BMI, PIR, sex, educational attainment, ethnicity, serum cotinine content, alcohol consumption, the presence or absence of diabetes and hypertension, eGFR, and dietary energy intake, amounting to a total of twelve items. These contained continuous and categorical variables, which were obtained by filling out questionnaires or laboratory tests. In the study, BMI was defined as weight divided by height squared and measured in kg/m^2^. The education levels were divided into high school education or below, high school education, and university degree and above. Ethnicities were divided into non-Hispanic white, non-Hispanic black, other Hispanic, and Mexican American or other. Alcohol consumption was based on the results of the questionnaire. The limit was 12 drinks per year, which was divided into less than 12 drinks per year and more than 12 drinks per year. Five indicators were selected to help determine whether there was diabetes or hypertension. If any of these items were met, the diagnosis could be made. Diabetes: Doctor said you have diabetes; glycohemoglobin content ≥6.5%; taking insulin now; fasting glucose ≥126 mg/dL; and taking diabetic pills to lower blood sugar. High blood pressure (HBP): Ever told you had HBP; taking a prescription for hypertension; now taking prescribed medicine for HBP; and systolic blood pressure average ≥140 mmHg and/or diastolic blood pressure average ≥90 mmHg. The formula for estimating glomerular filtration rate is eGFR = 141 × min(S_Cr_/κ,1)^α^ × max(S_Cr_/κ, 1)^−1.209^ × 0.993^age^ × 1.018 [if female] × 1.159 [if black], where S_Cr_ is the standardized serum creatinine in mg/dL, κ is 0.7 for women and 0.9 for men, a is −0.329 for women and −0.411 for men, min indicates the minimum of S_Cr_/κ or 1, and max indicates the maximum of S_Cr_/κ or 1. eGFR values are presented in mL/min/1.73 m^2^ [40].

### 2.5. Methods for Statistical Analysis

Data from each participant in the included study were collated. Dietary fiber intake was divided into four groups by quartile. Continuous variables following a normal distribution were expressed as mean ± standard deviations, and analysis of variance was used for comparisons between groups. If the data showed a skewed distribution, the median was used, with both 25th and 75th percentiles, and then compared by the Kruskal–Wallis rank-sum test. For categorical variables expressed as constituent ratios, the chi-squared test was used for data analysis.

In this study, serum Klotho levels were logarithmically converted so that they were treated as normally distributed. Multivariable linear regression models were used to estimate the association between dietary fiber intake and log-Klotho, with adjustment for potential covariates selected on the basis of relevant published literature [41,42,43,44,45,46,47,48]. In this study, three different models were constructed. Model 1 was a crude model. Model 2 was adjusted for age and sex. Model 3 was a fully adjusted model, which included multiple covariates (e.g., age, sex, BMI, PIR, educational attainment, ethnicity, serum cotinine content, alcohol consumption, diabetes or not, hypertension or not, eGFR, and dietary energy intake). To better interpret the results, we calculated the percentage changes and its 95% confidence intervals (CIs) according to regression coefficients, with the following conversion formula: (e^(IQR×β)^ − 1) × 100%, (e^[IQR×(β±1.96×SE)]^ − 1) × 100%. Furthermore, to explore a potential non-linear relationship between dietary fiber intake and serum Klotho level, we grouped dietary fiber intake into four categories based on its quartiles, and the categorical variable was included in models, with the lowest quartile as a reference. The percentage change and its 95% confidence intervals (CIs) were calculated from the regression coefficients using the following formula: (e^β^ − 1) × 100%, (e^(β±1.96×SE)^ − 1) × 100%. We thereafter performed tests for linear trends by entering the median value of each group of dietary fiber intake as a continuous variable in the models. In addition, we built restricted cubic spline (RCS) models to capture a potential non-linear dose–response relationship between dietary fiber intake and serum Klotho levels, with knots placed at 10th, 50th, and 90th and adjusted for the above-mentioned covariates.

Moreover, to explore the potential age, body shape, and sex-specific association between dietary fiber intake and serum Klotho levels, we further performed several subgroup analyses according to age (<60 years, ≥60 years), BMI (normal weight: <25, overweight and obesity: ≥25), and sex (women, men). We inserted the dietary fiber intake × age, dietary fiber intake × BMI, and dietary fiber intake × sex as interaction terms into models to obtain the *p* values for interaction. Similarly, we used RCS models to explore the non-linear dose–response relationship between dietary fiber intake and serum Klotho levels in each subgroup, with knots placed at 10th, 50th, and 90th and adjusted for the above-mentioned covariates.

The data collation and analysis used in the study were carried out using R software 4.2.2 version. We adopted the MEC weights recommended by the NHANES database to make the study population data nationally representative. A bilateral *p* < 0.05 was considered statistically significant.

## 3. Results

### 3.1. Basic Characteristics of All Participants

Table 1 summarizes clinical and biochemical data from the study population. A total of 11,282 participants (5544 (49.1%) males and 5738 (50.9%) females) were included in this study, with an average age of 57.8 ± 10.8 years. Their average BMI was 29.9 ± 6.7 kg/m^2^. Of these, more than half of the participants were college-educated and above, and nearly half were non-Hispanic white. The daily dietary fiber intake of the study participants ranged from 0 to 113.9 g, and their median serum Klotho level was 800.6 (653.9–990.0) pg/mL. Considering a comparison between quartile groups of dietary fiber intake, participants had significant differences with respect to BMI, PIR, serum cotinine, eGFR, and dietary energy intake, but not age. Also, the composition of participants’ sex, education, ethnicity, alcohol consumption, and the presence of diabetes and hypertension differed significantly between the quartiles of dietary fiber intake.

### 3.2. Association between Serum Klotho Levels and Dietary Fiber Intake

The association between dietary fiber intake and serum Klotho levels is presented in Table 2. A significant association was found between continuous dietary fiber intake and serum Klotho levels with or without adjustment for potential confounders. In Model 1, i.e., the crude model, serum Klotho levels increased by 1.5% (95% CI: 0.6%, 2.3%) for each interquartile range (IQR) increase in dietary fiber intake in all study participants. In Model 2, after adjusting for age and sex, each IQR increase in dietary fiber intake was associated with a 1.9% (95% CI: 1.0%, 2.8%) increase in serum Klotho levels. In Model 3, significant positive associations remained after further adjustment for the covariates of BMI, PIR, educational attainment, ethnicity, serum cotinine, alcohol consumption, diabetes, hypertension, eGFR, and dietary energy intake (percentage change: 1.9%; 95% CI: 0.8%, 3.0%). In the fully adjusted model, the percentage change in serum Klotho levels for participants in the second, third, and fourth quartiles of dietary fiber intake were 1.4% (95% CI: −1.2%, 4.1%), 3.1% (95% CI: 0.5%, 5.9%), and 4.7% (95% CI:1.8%, 7.6%), respectively, using the first quartile of dietary fiber intake as the reference (*p* for trend < 0.001).

**Figure 2:**
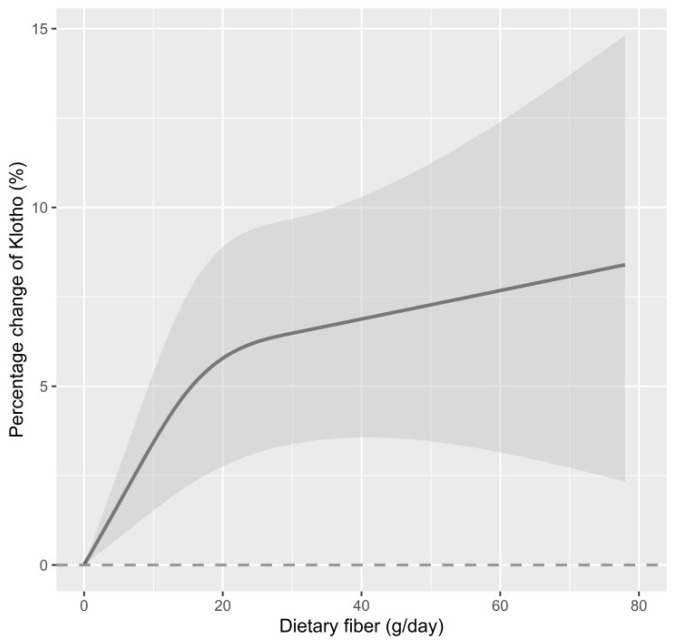
The dose–response relationship between dietary fiber intake and serum Klotho in all participants. Point value estimation (solid line) and 95% confidence interval calculation (dashed line) were estimated using a restrictive cubic spline analysis model, knotted at the 10th, 50th, and 90th percentiles (minimum value as reference). The model was adjusted for age, sex, BMI, PIR, education attainment, ethnicity, serum cotinine, alcohol drinking, diabetes, hypertension, eGFR, and dietary energy intake. *p* for non-linearity = 0.016.

Figure 2 illustrates our further exploration of the dose–response relationship between dietary fiber intake and serum Klotho levels using an RCS function. The results revealed a non-linear dose–response relationship between the two in all study participants (*p* for non-linearity = 0.016).

### 3.3. Subgroup Analysis

The results of subgroup analyses are presented in Table 3. After adjusting for potential confounders, we observed a significant association between dietary fiber intake and serum Klotho in the older, overweight and obese, and male and female groups. For the older group of participants, serum Klotho levels increased by 2.8% (95% CI: 1.3%, 4.4%) for each IQR increase in dietary fiber intake. In the overweight and obese population, each IQR increase in dietary fiber intake corresponded to a 1.4% (95% CI: 0.1%, 2.6%) increase in serum Klotho levels. In men, serum Klotho levels increased by 2.2% (95% CI: 1%, 3.4%) for each IQR increase in dietary fiber intake. In the female population, serum Klotho levels increased by 1.5% (95% CI: −0.3%, 3.3%) for each IQR increase in dietary fiber intake. When dietary fiber intake was considered as a categorical variable, compared with participants in the lowest quartile of dietary fiber intake, serum Klotho levels increased by 7.0% (95% CI: 2.5%, 11.7%), 4.9% (95% CI: 1.5%, 8.4%), 4.7% (95% CI: 0.3%, 9.3%), and 4.6% (95% CI: 0.6%, 8.7%) in the highest quartile of the elderly, overweight and obese, and male and female subgroups, respectively. An RCS model was used to determine the dose–response relationship between dietary fiber intake and serum Klotho levels in each subgroup (see Figure S1). The results displayed a linear dose–response relationship between the two in the older group of participants (older: *p* for non-linearity = 0.097). Conversely, a non-linear dose–response relationship was observed between dietary fiber intake and serum Klotho levels in the overweight and obese groups, as well as male and female groups (overweight and obesity: *p* for non-linearity = 0.005, male: *p* for non-linearity = 0.024, female: *p* for non-linearity = 0.014).

## 4. Discussion

In this study, we explored the association between dietary fiber intake and serum Klotho levels in data from a representative US-wide population containing 11,282 participants. The results revealed a significant, positive correlation between dietary fiber intake and serum Klotho levels in all participants, displaying a significant non-linearity dose–response relationship. Additionally, the relationship between dietary fiber intake and Klotho was more prominent in older and overweight and obese participants.

A growing body of evidence suggests that Klotho levels are associated with multiple diseases and are involved in the pathogenesis of chronic diseases [49]. Several lines of evidence have shown that lifestyle, especially dietary habits and patterns, may affect serum Klotho levels. For instance, a small sample intervention study (N = 44) found that a high-salt diet could significantly reduce serum Klotho levels [50]. Another intervention study of patients with chronic kidney disease (N = 79) demonstrated that those with high dietary phosphorus intake had significantly lower levels of Klotho than those with low dietary phosphorus intake [51]. More recently, Ma et al. showed a significant correlation between pro-inflammatory diets and reduced blood Klotho levels in middle-aged and elderly populations using data from the NHANES [52]. A similar association between pro-inflammatory diets and Klotho was observed among middle-aged sedentary adults, suggesting the potential role of anti-inflammatory nutrients in regulating Klotho [53]. Dietary fiber, a common antioxidant nutrient, has been demonstrated to have significant antiaging benefits, with people who consume high levels of dietary fiber living longer than their peers and having a reduced risk of disease or premature death. However, little is known about the relationship between dietary fiber intake and serum Klotho levels. To the best of our knowledge, there has been only one study addressing this issue. Ostojic et al. found a positive correlation between dietary fiber intake and Klotho levels, but this association became non-significant after adjusting for gender and age (N = 2637), which is different from the present study [36]. Although both studies utilized the NHANES data, differences in the sample sizes, confounders, and sampling weights used in regression models might explain the inconsistent results. In the present study, NHANES data from 2007–2008 to 2015–2016 were combined (N = 11,282), and multivariable models with survey weighting procedures were carried out. Notably, the present study further explored a dose–response relationship between dietary fiber intake and Klotho, and results showed a significant, non-linear relationship. Our findings extend the limited available evidence on the effects of various dietary factors on Klotho levels. Based on the dose–response curve in this study, it can be observed that when dietary fiber intake was below 20 g/day, even a slight increase in dietary fiber was significantly related to Klotho levels. However, after reaching 20 g/day, the rate of Klotho increase slowed down with further dietary fiber intake. Although the detailed mechanism is difficult to speculate, this result implies that from an individual perspective, even a slight supplementation of dietary fiber can contribute to the enhancement of Klotho levels for those who have particularly low daily dietary fiber intake.

In addition to population-based studies, there were several experimental findings that echo the results of our analysis. It is well known that Klotho is an aging-related marker [20]. Yu et al. asserted that characteristic dietary fiber compounds had a maintenance and improvement effect on learning and memory ability in aging mice, as well as improved antioxidant capacity and reduced inflammation levels. This implies that dietary fiber complexes have antiaging effects in naturally aging mice [54]. In another example, supplementation with prebiotic high-esterified pectin in rats was found to improve blood pressure, reduce cardiac lipid content, and increase circulating levels of fibroblast growth factor 21 and its co-receptor Klotho expression [55]. As reported by Wang et al., the intervention of sugarcane fiber induced hepatic Klotho expression and AMPK signaling pathway activation in mice [56].

The specific molecular mechanisms through which dietary fiber intake affects serum Klotho levels have not been fully elucidated. The role of dietary fiber in anti-inflammatory and antioxidant properties might shed light on potential mechanisms. As reported, inflammation significantly downregulates Klotho protein expression [28]. In contrast, dietary fiber can be involved in improving chronic systemic inflammation by interacting with the gut microbiome. Studies have underlined the fact that dietary fiber intake directly affects the number of Clostridiales in the gut microbiome, which plays a key role in the regulation of local and systemic inflammation [57]. The results of many epidemiological studies also confirm that increased dietary fiber intake is associated with a reduced risk of developing several chronic inflammatory diseases [58]. This suggests that increased dietary fiber intake may be beneficial in antagonizing the reduction in Klotho levels induced by inflammation. On the other hand, oxidative stress has been demonstrated to play a key role in downregulating Klotho levels [35]. Dietary fiber inhibits oxidative stress through its metabolites, short-chain fatty acids, among which the levels of acetate, propionate, and butyrate are high. Butyrate, in particular, acts as an antioxidant by regulating oxidoreductase activity, activating nuclear factor erythroid 2-related factor 2 transcription, and preventing the production of reactive oxygen species and reactive nitrogen species [59]. Therefore, the antioxidant effect of dietary fiber may play a role in the maintenance of stable Klotho levels. Additionally, as Marsell et al. have demonstrated, FGF23 exerts a significant inhibitory effect on Klotho expression. Their study involved gene expression microarray analysis of the kidneys of transgenic mice overexpressing FGF23, revealing the most pronounced decrease in Klotho mRNA among all the analyzed transcripts [60]. Furthermore, a population-based study revealed a significant impact of increased dietary fiber intake on reducing FGF23 levels [61]. Therefore, the downregulation of FGF23 may also play a crucial role in the association between dietary fiber and Klotho. However, further animal studies or longitudinal cohort studies are still required to elucidate the detailed specific mechanisms.

Previous studies have proven that Klotho levels decrease with age [41]. Therefore, we performed a subgroup analysis stratified by age. In the stratified results, increased dietary fiber intake was associated with elevated serum Klotho levels in older participants, but no significant association was found in middle-aged participants. The possible reason for this phenomenon may be related to the different intestinal microbiota between the older and middle-aged participants. Aging is accompanied by changes in the intestinal microbiota. A study found that the ratios of *Firmicutes* to *Bacteroidetes* and the bacteria producing short-chain fatty acids in the elderly population were significantly decreased compared with the younger population [62,63]. One important way in which dietary fiber produces positive health effects is by modulating intestinal microorganisms, such as lowering *Bacteroidetes* and upregulating *Lactobacillus* and *Bifidobacterium* [64,65], which then exert anti-inflammatory and antioxidant effects. Thus, in the elderly population, dietary fiber intake may impact the gut microbiota and reshape its composition, affecting the regulation of Klotho levels and insulating Klotho expression from the increased levels of inflammation and oxidative stress in the body. However, as the interaction was not statistically significant, the association between dietary fiber intake and serum Klotho levels should be viewed with caution for differences between middle and older subgroups.

Numerous population studies have shown that BMI is associated with serum Klotho levels [66]. Similarly, we analyzed the relationship between dietary fiber intake and serum Klotho levels in different BMI subgroups. The result indicates that increased dietary fiber intake was associated with elevated serum Klotho levels in overweight and obese participants, but no similar association was found in normal-weight participants. This may be attributed to the heightened requirement of dietary fiber in overweight/obese individuals, in order to address the challenges posed by metabolic disorders or chronic inflammation. Consequently, individuals with overweight/obesity may have a greater dependence on a higher intake of dietary fiber to facilitate the production of Klotho protein. In addition, considering the elevated disease burden associated with overweight/obesity, the weight status-specific findings might help to develop specific dietary guidance and nutrition education plans for individuals with obesity, encouraging increased consumption of dietary fiber. These interventions are anticipated to mitigate chronic inflammation levels, enhance metabolic health, and ultimately diminish the susceptibility to obesity-related diseases. While the significant interaction effect between weight status and dietary fiber intake on Klotho levels was observed, it is important to interpret these associations cautiously due to the cross-sectional study design. Further longitudinal studies are still warranted to validate the findings presented in this study.

Although this study consists of a nationally representative middle-aged and elderly population and encompasses comprehensive covariate information, the relatively large sample size in this study enabled the exploration of multiple subgroup analyses. However, there are several shortcomings in the study. First, as a cross-sectional study, it cannot account for the causal or temporal association between the two factors studies. Second, the NHANES database contains serum Klotho data only for participants aged 40–79 years. It is uncertain whether the results obtained are equally applicable to the young population younger than 40 years. Third, only dietary fiber intake data from the first 24 h dietary review were used in the study, which may have impacted evaluation accuracy due to the natural day-to-day variability in dietary preferences. Fourth, the differences in seasonal dietary preferences might have a potential impact on the findings of this study. Finally, although we adjusted several covariates to eliminate their effects, there may still be unknown confounding factors that we have not yet identified. Continued attention and interpretation of the association between dietary fiber intake and serum Klotho levels remains an important topic for the future.

## 5. Conclusions

In summary, the findings of this study provide evidence of a positive association between dietary fiber intake and increased levels of Klotho protein. As the Klotho protein is closely related to aging-related diseases, including cardiovascular disease and chronic kidney disease, increasing dietary fiber intake may have important implications for promoting healthy aging and reducing the risk of these diseases. Future research should confirm these findings through prospective studies and explore other potential health benefits of dietary fiber. Additionally, if these results are verified through prospective and/or intervention studies, patients with aging-related diseases may greatly benefit from increasing Klotho protein levels through increased dietary fiber intake.

## Figures and Tables

**Table 1:** Baseline characteristics of participants in NHANES (N = 11,282).

Variables	Total Participants	Dietary Fiber Intake (g/Day)				
		Quartile 1	Quartile 2	Quartile 3	Quartile 4	*p* Value
Age, years, mean (SD)	57.8 (10.8)	57.6 (10.8)	58.2 (11.0)	57.8 (10.9)	57.5 (10.5)	0.059
BMI, kg/m^2^, mean (SD)	29.9 (6.7)	30.3 (7.3)	30.1 (6.8)	29.7 (6.4)	29.4 (6.4)	<0.001
PIR, mean (SD)	2.7 (1.7)	2.3 (1.6)	2.6 (1.6)	2.8 (1.7)	2.9 (1.7)	<0.001
Sex, *n* (%)						<0.001
male	5544 (49.1)	1141 (40.7)	1254 (44.4)	1410 (49.8)	1739 (61.6)	
female	5738 (50.9)	1663 (59.3)	1571 (55.6)	1421 (50.2)	1083 (38.4)	
Educational attainment, *n* (%)						<0.001
<High school	2950 (26.1)	883 (31.5)	682 (24.1)	681 (24.1)	704 (24.9)	
High school	2519 (22.3)	716 (25.5)	731 (25.9)	587 (20.7)	485 (17.2)	
College or above	5813 (51.5)	1205 (43.0)	1412 (50.0)	1563 (55.2)	1633 (57.9)	
Race/Ethnicity, *n* (%)						<0.001
Non-Hispanic white	5203 (46.1)	1263 (45.0)	1372 (48.6)	1331 (47.0)	1237 (43.8)	
Non-Hispanic black	2234 (19.8)	760 (27.1)	608 (21.5)	463 (16.4)	403 (14.3)	
Other Hispanic	1199 (10.6)	300 (10.7)	316 (11.2)	320 (11.3)	263 (9.3)	
Mexican American or other	2646 (23.5)	481 (17.2)	529 (18.7)	717 (25.3)	919 (32.6)	
Serum cotinine, ng/mL, median (25th–75th)	0.0 (0.0–2.5)	0.1 (0.0–179.0)	0.0 (0.0–1.7)	0.0 (0.0–0.3)	0.0 (0.0–0.1)	<0.001
Alcohol drinking, *n* (%)						<0.001
More than 12 drinks/yr	3199 (28.4)	864 (30.8)	853 (30.2)	778 (27.5)	704 (24.9)	
Less than 12 drinks/yr	8083 (71.6)	1940 (69.2)	1972 (69.8)	2053 (72.5)	2118 (75.1)	
Diabetes, *n* (%)						0.002
no	8569 (76.0)	2076 (74.0)	2118 (75.0)	2173 (76.8)	2202 (78.0)	
yes	2713 (24.0)	728 (26.0)	707 (25.0)	658 (23.2)	620 (22.0)	
Hypertension, *n* (%)						<0.001
no	5133 (45.5)	1155 (41.2)	1216 (43.0)	1337 (47.2)	1425 (50.5)	
yes	6149 (54.5)	1649 (58.8)	1609 (57.0)	1494 (52.8)	1397 (49.5)	
eGFR, mL/min/1.73 m^2^, mean (SD)	84.0 (19.6)	82.2 (21.0)	83.0 (20.1)	84.5 (19.0)	86.4 (18.2)	<0.001
Dietary energy intake, kcal/day, mean (SD)	2023.7 (914.1)	1431.4 (619.2)	1864.0 (713.7)	2139.3 (759.5)	2655.9 (1041.1)	<0.001
Serum Klotho, pg/mL, median (25th–75th)	800.6 (653.9–990.0)	783.8 (631.2–977.4)	800.5 (657.3–987.1)	805.6 (659.2–991.9)	810.9 (662.0–1002.6)	0.002

Note: SD, standard deviation; BMI, body mass index; PIR, family poverty income ratio; eGFR, estimated glomerular filtration rate. Dietary fiber intake quartile ranges: Quartile 1 = 0.0 to 9.6 g/day; Quartile 2 = 9.7 to 14.8 g/day; Quartile 3 = 14.9 to 21.7 g/day; Quartile 4: 21.8 to 113.9 g/day.

**Table 2:** The association between dietary fiber intake and serum Klotho levels among all participants.

Dietary Fiber (g/Day)	Percent Changes (%) and 95% CI					
	Model 1	*p* Value	Model 2	*p* Value	Model 3	*p* Value
Per IQR increases	1.5 (0.6, 2.3)	0.001	1.9 (1.0, 2.8)	<0.001	1.9 (0.8, 3.0)	<0.001
Quartile 1	Ref		Ref		Ref	
Quartile 2	0.9 (−1.5, 3.3)	0.489	1.3 (−1.1, 3.8)	0.283	1.4 (−1.2, 4.1)	0.311
Quartile 3	2.5 (0.1, 5.0)	0.043	3.2 (0.8, 5.6)	0.011	3.1 (0.5, 5.9)	0.025
Quartile 4	3.5 (1.3, 5.7)	0.002	4.6 (2.3, 6.9)	<0.001	4.7 (1.8, 7.6)	0.002
*p* for trend		<0.001		<0.001		<0.001

Note: CI, confidence interval; IQR, interquartile range; Model 1 was the crude model, including dietary fiber intake; Model 2 was adjusted for age and sex; Model 3 was further adjusted for BMI, PIR, education attainment, ethnicity, serum cotinine, alcohol drinking, diabetes or not, hypertension or not, eGFR and dietary energy intake. Dietary fiber intake quartile ranges: Quartile 1 = 0 to 9.6 g/day; Quartile 2 = 9.7 to 14.8 g/day; Quartile 3 = 14.9 to 21.7 g/day; Quartile 4: 21.8 to 113.9 g/day.

**Table 3:** The relationship between dietary fiber intake and serum Klotho levels, stratified by age, BMI, and sex.

Participants	Dietary Fiber Intake (g/Day)	Percent Changes (%) and 95% CI	*p* Value	*p* ^a^ for Interaction
Age subgroup				0.074
Age < 60 years	Per IQR increases	1.3 (−0.1, 2.7)	0.077	
	Quartile 1	Ref		
	Quartile 2	0.6 (−2.8, 4.2)	0.728	
	Quartile 3	0.7 (−2.2, 3.7)	0.634	
	Quartile 4	3.1 (−0.6, 7.0)	0.107	
	*p* for trend		0.077	
Age ≥ 60 years	Per IQR increases	2.8 (1.3, 4.4)	0.001	
	Quartile 1	Ref		
	Quartile 2	2.7 (−1.1, 6.6)	0.175	
	Quartile 3	7.5 (2.5, 12.7)	0.004	
	Quartile 4	7.0 (2.5, 11.7)	0.003	
	*p* for trend		0.002	
BMI subgroup				0.036
BMI < 25 kg/m^2^	Per IQR increases	3.1 (1.0, 5.3)	0.005	
	Quartile 1	Ref		
	Quartile 2	−4.2 (−8.9, 0.7)	0.098	
	Quartile 3	0.2 (−4.5, 5.1)	0.929	
	Quartile 4	2.6 (−3.0, 8.4)	0.372	
	*p* for trend		0.069	
BMI ≥ 25 kg/m^2^	Per IQR increases	1.4 (0.1, 2.6)	0.036	
	Quartile 1	Ref		
	Quartile 2	3.0 (0.0, 6.1)	0.057	
	Quartile 3	3.9 (0.5, 7.4)	0.027	
	Quartile 4	4.9 (1.5, 8.4)	0.006	
	*p* for trend		0.007	
Sex subgroup				0.252
male	Per IQR increases	2.2 (1.0, 3.4)	0.001	
	Quartile 1	Ref		
	Quartile 2	−0.7 (−4.8, 3.6)	0.742	
	Quartile 3	3.4 (−0.6, 7.7)	0.104	
	Quartile 4	4.7 (0.3, 9.3)	0.039	
	*p* for trend		0.003	
female	Per IQR increases	1.5 (−0.3, 3.3)	0.106	
	Quartile 1	Ref		
	Quartile 2	3.1 (0.2, 6.1)	0.041	
	Quartile 3	3.1 (−0.1, 6.3)	0.064	
	Quartile 4	4.6 (0.6, 8.7)	0.026	
	*p* for trend		0.052	

Note: CI, confidence interval; IQR, interquartile range; BMI, body mass index. Dietary fiber intake quartile ranges: Quartile 1 = 0 to 9.6 g/day; Quartile 2 = 9.7 to 14.8 g/day; Quartile 3 = 14.9 to 21.7 g/day; Quartile 4: 21.8 to 113.9 g/day. The age subgroup analysis adjusted for the following covariates: sex, BMI, PIR, education attainment, ethnicity, serum cotinine, alcohol drinking, diabetes or not, hypertension or not, eGFR, and dietary energy intake. The BMI subgroup analysis adjusted for the following covariates: age, sex, PIR, education attainment, ethnicity, serum cotinine, alcohol drinking, diabetes or not, hypertension or not, eGFR, and dietary energy intake. The gender subgroup analysis adjusted for the following covariates: age, BMI, PIR, education attainment, ethnicity, serum cotinine, alcohol drinking, diabetes or not, hypertension or not, eGFR, and dietary energy intake. ^a^ *p* value for the interaction of dietary fiber intake with age, BMI, or sex.

## Data Availability

The datasets generated and analyzed during the current study are publicly available from the NHANES website (https://wwwn.cdc.gov/nchs/nhanes/Default.aspx, accessed on 18 March 2023).

## Supplementary Materials

The following supporting information can be downloaded at: https://www.mdpi.com/article/10.3390/nu15143147/s1, Figure S1. The dose–response relationships between dietary fiber intake and serum Klotho in various subgroups. Table S1. Datasets used in analysis.
