# Supplementary material for: The Association between Dietary Fiber Intake and Serum Klotho Levels in Americans: A Cross-Sectional Study from the National Health and Nutrition Examination Survey
Source: Nutrients. 2023 Jul 14;15(14):3147. doi: 10.3390/nu15143147 (PMC10385840; doi:10.3390/nu15143147)
Supplement: Supplementary file 1 [file nutrients-15-03147-s001.zip › Table S1.pdf]

Table S1. Data sets used in analysis

Variables	Variable name in NHANES	Data file name in NHANES	Years	Links	Data types	Unit/ Component ratio
Age	ridageyr	DEMO_E	2007-2008	https://wwwn.cdc.gov/nchs/nhanes/search/datapage.aspx?Component=Demographics&CycleBeginYear=2007	Continuous variable	years; mean (SD)
		DEMO_F	2009-2010	https://wwwn.cdc.gov/nchs/nhanes/search/datapage.aspx?Component=Demographics&CycleBeginYear=2009		
		DEMO_G	2011-2012	https://wwwn.cdc.gov/nchs/nhanes/search/datapage.aspx?Component=Demographics&CycleBeginYear=2011		
		DEMO_H	2013-2014	https://wwwn.cdc.gov/nchs/nhanes/search/datapage.aspx?Component=Demographics&CycleBeginYear=2013		
		DEMO_I	2015-2016	https://wwwn.cdc.gov/nchs/nhanes/search/datapage.aspx?Component=Demographics&CycleBeginYear=2015		
PIR	indfmpir	DEMO_E	2007-2008	https://wwwn.cdc.gov/nchs/nhanes/search/datapage.aspx?Component=Demographics&CycleBeginYear=2007	Continuous variable	/; mean (SD)
		DEMO_F	2009-2010	https://wwwn.cdc.gov/nchs/nhanes/search/datapage.aspx?Component=Demographics&CycleBeginYear=2009		
		DEMO_G	2011-2012	https://wwwn.cdc.gov/nchs/nhanes/search/datapage.aspx?Component=Demographics&CycleBeginYear=2011		
		DEMO_H	2013-2014	https://wwwn.cdc.gov/nchs/nhanes/search/datapage.aspx?Component=Demographics&CycleBeginYear=2013		
		DEMO_I	2015-2016	https://wwwn.cdc.gov/nchs/nhanes/search/datapage.aspx?Component=Demographics&CycleBeginYear=2015		
Sex	riagendr	DEMO_E	2007-2008	https://wwwn.cdc.gov/nchs/nhanes/search/datapage.aspx?Component=Demographics&CycleBeginYear=2007	Categorical variable	male, female; n (%)
		DEMO_F	2009-2010	https://wwwn.cdc.gov/nchs/nhanes/search/datapage.aspx?Component=Demographics&CycleBeginYear=2009		
		DEMO_G	2011-2012	https://wwwn.cdc.gov/nchs/nhanes/search/datapage.aspx?Component=Demographics&CycleBeginYear=2011		

		DEMO_H	2013-2014	https://wwwn.cdc.gov/nchs/nhanes/search/datapage.aspx?Component=Demographics&CycleBeginYear=2013		
		DEMO_I	2015-2016	https://wwwn.cdc.gov/nchs/nhanes/search/datapage.aspx?Component=Demographics&CycleBeginYear=2015		
Educational attainment	dmdeduc2	DEMO_E	2007-2008	https://wwwn.cdc.gov/nchs/nhanes/search/datapage.aspx?Component=Demographics&CycleBeginYear=2007	Categorical variable	<high school, high school, college or above; n (%)
		DEMO_F	2009-2010	https://wwwn.cdc.gov/nchs/nhanes/search/datapage.aspx?Component=Demographics&CycleBeginYear=2009		
		DEMO_G	2011-2012	https://wwwn.cdc.gov/nchs/nhanes/search/datapage.aspx?Component=Demographics&CycleBeginYear=2011		
		DEMO_H	2013-2014	https://wwwn.cdc.gov/nchs/nhanes/search/datapage.aspx?Component=Demographics&CycleBeginYear=2013		
		DEMO_I	2015-2016	https://wwwn.cdc.gov/nchs/nhanes/search/datapage.aspx?Component=Demographics&CycleBeginYear=2015		
Race/Ethnicity	ridreth1	DEMO_E	2007-2008	https://wwwn.cdc.gov/nchs/nhanes/search/datapage.aspx?Component=Demographics&CycleBeginYear=2007	Categorical variable	non Hispanic White, non Hispanic Black, other Hispanic, Mexican American or Other; n (%)
		DEMO_F	2009-2010	https://wwwn.cdc.gov/nchs/nhanes/search/datapage.aspx?Component=Demographics&CycleBeginYear=2009		
		DEMO_G	2011-2012	https://wwwn.cdc.gov/nchs/nhanes/search/datapage.aspx?Component=Demographics&CycleBeginYear=2011		
		DEMO_H	2013-2014	https://wwwn.cdc.gov/nchs/nhanes/search/datapage.aspx?Component=Demographics&CycleBeginYear=2013		
		DEMO_I	2015-2016	https://wwwn.cdc.gov/nchs/nhanes/search/datapage.aspx?Component=Demographics&CycleBeginYear=2015		
BMI	bmxbmi	BMX_E	2007-2008	https://wwwn.cdc.gov/nchs/nhanes/search/datapage.aspx?Component=Examination&CycleBeginYear=2007	Continuous variable	kg/m^2; mean (SD)
		BMX_F	2009-2010	https://wwwn.cdc.gov/nchs/nhanes/search/datapage.aspx?Component=Examination&CycleBeginYear=2009		
		BMX_G	2011-2012	https://wwwn.cdc.gov/nchs/nhanes/search/datapage.aspx?Component=Examination&CycleBeginYear=2011		

		BMX_H	2013-2014	https://wwwn.cdc.gov/nchs/nhanes/search/datapage.aspx?Component=Examination&CycleBeginYear=2013		
		BMX_I	2015-2016	https://wwwn.cdc.gov/nchs/nhanes/search/datapage.aspx?Component=Examination&CycleBeginYear=2015		
Dietary fiber intake	dr1tfibe	DR1IFF_E	2007-2008	https://wwwn.cdc.gov/nchs/nhanes/search/datapage.aspx?Component=Dietary&CycleBeginYear=2007	Continuous variable	g/day; median (25th–75th)
		DR1IFF_F	2009-2010	https://wwwn.cdc.gov/nchs/nhanes/search/datapage.aspx?Component=Dietary&CycleBeginYear=2009		
		DR1IFF_G	2011-2012	https://wwwn.cdc.gov/nchs/nhanes/search/datapage.aspx?Component=Dietary&CycleBeginYear=2011		
		DR1IFF_H	2013-2014	https://wwwn.cdc.gov/nchs/nhanes/search/datapage.aspx?Component=Dietary&CycleBeginYear=2013		
		DR1IFF_I	2015-2016	https://wwwn.cdc.gov/nchs/nhanes/search/datapage.aspx?Component=Dietary&CycleBeginYear=2015		
Dietary energy intake	dr1tkcal	DR1IFF_E	2007-2008	https://wwwn.cdc.gov/nchs/nhanes/search/datapage.aspx?Component=Dietary&CycleBeginYear=2007	Continuous variable	kcal/day; mean (SD)
		DR1IFF_F	2009-2010	https://wwwn.cdc.gov/nchs/nhanes/search/datapage.aspx?Component=Dietary&CycleBeginYear=2009		
		DR1IFF_G	2011-2012	https://wwwn.cdc.gov/nchs/nhanes/search/datapage.aspx?Component=Dietary&CycleBeginYear=2011		
		DR1IFF_H	2013-2014	https://wwwn.cdc.gov/nchs/nhanes/search/datapage.aspx?Component=Dietary&CycleBeginYear=2013		
		DR1IFF_I	2015-2016	https://wwwn.cdc.gov/nchs/nhanes/search/datapage.aspx?Component=Dietary&CycleBeginYear=2015		
Serum cotinine	lbxcot	COTNAL_E	2007-2008	https://wwwn.cdc.gov/nchs/nhanes/search/datapage.aspx?Component=Laboratory&CycleBeginYear=2007	Continuous variable	ng/mL; median (25th–75th)
		COTNAL_F	2009-2010	https://wwwn.cdc.gov/nchs/nhanes/search/datapage.aspx?Component=Laboratory&CycleBeginYear=2009		
		COTNAL_G	2011-2012	https://wwwn.cdc.gov/nchs/nhanes/search/datapage.aspx?Component=Laboratory&CycleBeginYear=2011		

		COT_H	2013-2014	https://wwwn.cdc.gov/nchs/nhanes/search/datapage.aspx?Component=Laboratory&CycleBeginYear=2013		
		COT_I	2015-2016	https://wwwn.cdc.gov/nchs/nhanes/search/datapage.aspx?Component=Laboratory&CycleBeginYear=2015		
Acohol drinking	alq101	ALQ_E	2007-2008	https://wwwn.cdc.gov/nchs/nhanes/search/datapage.aspx?Component=Questionnaire&CycleBeginYear=2007	Categorical variable	more than 12 drinks/yr, less than 12 drinks/yr; n (%)
		ALQ_F	2009-2010	https://wwwn.cdc.gov/nchs/nhanes/search/datapage.aspx?Component=Questionnaire&CycleBeginYear=2009		
		ALQ_G	2011-2012	https://wwwn.cdc.gov/nchs/nhanes/search/datapage.aspx?Component=Questionnaire&CycleBeginYear=2011		
		ALQ_H	2013-2014	https://wwwn.cdc.gov/nchs/nhanes/search/datapage.aspx?Component=Questionnaire&CycleBeginYear=2013		
		ALQ_I	2015-2016	https://wwwn.cdc.gov/nchs/nhanes/search/datapage.aspx?Component=Questionnaire&CycleBeginYear=2015		
Diabetes	diq010,lbxgh,diq050,lbxglu,diq070	GHB_E<br>GLU_E<br>DIQ_E	2007-2008	https://wwwn.cdc.gov/nchs/nhanes/search/datapage.aspx?Component=Laboratory&CycleBeginYear=2007<br>https://wwwn.cdc.gov/nchs/nhanes/search/datapage.aspx?Component=Questionnaire&CycleBeginYear=2007	Categorical variable	no, yes; n (%)
		GHB_F<br>GLU_F<br>DIQ_F	2009-2010	https://wwwn.cdc.gov/nchs/nhanes/search/datapage.aspx?Component=Laboratory&CycleBeginYear=2009<br>https://wwwn.cdc.gov/nchs/nhanes/search/datapage.aspx?Component=Questionnaire&CycleBeginYear=2009		
		GHB_G<br>GLU_G<br>DIQ_G	2011-2012	https://wwwn.cdc.gov/nchs/nhanes/search/datapage.aspx?Component=Laboratory&CycleBeginYear=2011<br>https://wwwn.cdc.gov/nchs/nhanes/search/datapage.aspx?Component=Questionnaire&CycleBeginYear=2011		
		GHB_H<br>GLU_H<br>DIQ_H	2013-2014	https://wwwn.cdc.gov/nchs/nhanes/search/datapage.aspx?Component=Laboratory&CycleBeginYear=2013<br>https://wwwn.cdc.gov/nchs/nhanes/search/datapage.aspx?Component=Questionnaire&CycleBeginYear=2013		
		GHB_I<br>GLU_I	2015-2016	https://wwwn.cdc.gov/nchs/nhanes/search/datapage.aspx?Component=Laboratory&CycleBeginYear=2015<br>https://wwwn.cdc.gov/nchs/nhanes/search/datapage.aspx?Component=Questionnaire&CycleBeginYear=2015		

		DIQ_I				
Hypertension	bpxsy1,bpxsy2,bpxsy3,bpxsy4,<br>bpxdi1,bpxdi2,bpxdi3,bpxdi4,<br>bpq020,bpq040a,bpq050a	BPX_E<br>BPQ_E	2007-2008	https://wwwn.cdc.gov/nchs/nhanes/search/datapage.aspx?Component=Examination&CycleBeginYear=2007<br>https://wwwn.cdc.gov/nchs/nhanes/search/datapage.aspx?Component=Questionnaire&CycleBeginYear=2007	Categorical variable	no, yes; n (%)
		BPX_F<br>BPQ_F	2009-2010	https://wwwn.cdc.gov/nchs/nhanes/search/datapage.aspx?Component=Examination&CycleBeginYear=2009<br>https://wwwn.cdc.gov/nchs/nhanes/search/datapage.aspx?Component=Questionnaire&CycleBeginYear=2009		
		BPX_G<br>BPQ_G	2011-2012	https://wwwn.cdc.gov/nchs/nhanes/search/datapage.aspx?Component=Examination&CycleBeginYear=2011<br>https://wwwn.cdc.gov/nchs/nhanes/search/datapage.aspx?Component=Questionnaire&CycleBeginYear=2011		
		BPX_H<br>BPQ_H	2013-2014	https://wwwn.cdc.gov/nchs/nhanes/search/datapage.aspx?Component=Examination&CycleBeginYear=2013<br>https://wwwn.cdc.gov/nchs/nhanes/search/datapage.aspx?Component=Questionnaire&CycleBeginYear=2013		
		BPX_I<br>BPQ_I	2015-2016	https://wwwn.cdc.gov/nchs/nhanes/search/datapage.aspx?Component=Examination&CycleBeginYear=2015<br>https://wwwn.cdc.gov/nchs/nhanes/search/datapage.aspx?Component=Questionnaire&CycleBeginYear=2015		
eGFR	lbdscrsi	BIOPRO_E	2007-2008	https://wwwn.cdc.gov/nchs/nhanes/search/datapage.aspx?Component=Laboratory&CycleBeginYear=2007	Continuous variable	mL/min/1.73 m^2; mean (SD)
		BIOPRO_F	2009-2010	https://wwwn.cdc.gov/nchs/nhanes/search/datapage.aspx?Component=Laboratory&CycleBeginYear=2009		
		BIOPRO_G	2011-2012	https://wwwn.cdc.gov/nchs/nhanes/search/datapage.aspx?Component=Laboratory&CycleBeginYear=2011		
		BIOPRO_H	2013-2014	https://wwwn.cdc.gov/nchs/nhanes/search/datapage.aspx?Component=Laboratory&CycleBeginYear=2013		
		BIOPRO_I	2015-2016	https://wwwn.cdc.gov/nchs/nhanes/search/datapage.aspx?Component=Laboratory&CycleBeginYear=2015		
serum Klotho	sskloth	SSKL_E	2007-2008	https://wwwn.cdc.gov/nchs/nhanes/search/datapage.aspx?Component=Laboratory&CycleBeginYear=2007	Continuous variable	pg/mL; median (25th–75th)
		SSKL_F	2009-2010	https://wwwn.cdc.gov/nchs/nhanes/search/datapage.aspx?Component=Laboratory&CycleBeginYear=2009		
		SSKL_G	2011-2012	https://wwwn.cdc.gov/nchs/nhanes/search/datapage.aspx?Component=Laboratory&CycleBeginYear=2011		
		SSKL_H	2013-2014	https://wwwn.cdc.gov/nchs/nhanes/search/datapage.aspx?Component=Laboratory&CycleBeginYear=2013		

		SSKL_I	2015-2016	https://wwwn.cdc.gov/nchs/nhanes/search/datapage.aspx?Component=Laboratory&CycleBeginYear=2015		
